# Multiple osseous involvements in a case of disseminated cryptococcosis

**DOI:** 10.4103/0019-5413.65158

**Published:** 2010

**Authors:** Rakesh Singh, I Xess

**Affiliations:** Department of Microbiology, All India Institute of Medical Sciences, New Delhi, India

**Keywords:** Disseminated cryptococcosis, vertebral cryptococcosis, *Cryptococcus neoformans*

## Abstract

Osseous involvement occurs in 5–10% of patients with disseminated cryptococcosis. We are reporting an unusual case of disseminated cryptococcosis involving the sternum and lumbar vertebra with the formation of psoas abscess with pulmonary tuberculosis. The patient presented with fever for 3 months. A diagnosis of pulmonary tuberculosis was made on thoracic contrast-enhanced computerized tomography and she was put on antituberculosis treatment. She was immunocompetent with negative human immunodeficiency virus. She conceived subsequently and had complaints of backache and swelling over the sternum. Magnetic resonance imaging showed destruction of L5 vertebra with psoas abscess. Vertebral cryptococcosis may mimic tuberculosis and malignancy. She had a bad obstetric history and experienced five, first-trimester spontaneous abortions in each successive year since 2001. This pregnancy again resulted in spontaneous abortion. *Cryptococcus neoformans* was isolated from two different sites: pus-involving the sternum and ultrasound-guided psoas abscess aspirate. Serum latex agglutination test for cryptococcal capsular polysaccharide antigen was positive. The diagnosis of cryptococcosis was delayed because the patient was diagnosed as a case of pulmonary tuberculosis, wherein clinical signs, symptoms and radiological findings in both the conditions are similar. Amphotericin B was started but she developed varicella infection and expired due to cardiac failure.

## INTRODUCTION

Cryptococcosis is caused by *Cryptococcus neoformans*. It is 4–6-*µ* encapsulated yeast and present ubiquitously in nature, especially in soil contaminated with pigeon fecal excreta. The organism enters into the human body through the lung and then spreads to the central nervous system. It causes a wide spectrum of disease, from an asymptomatic pulmonary lesion to a fatal disseminated cryptococcosis. It has a potential for reactivation whenever opportunity for immunosuppression arises. The predisposing factors are advanced human immunodeficiency virus (HIV) stage and other conditions like prolonged use of corticosteroid, lymphomas, solid organ transplant recipients and patients with immune suppressive disease or receiving such drugs. Cryptococcosis occurs in 2.5–10% of all HIV-infected patients, with a mortality of 50% from India.^1^ Osseous involvement occurs in 5–10% of patients with disseminated cryptococcosis. The radiological findings and clinical features of cryptococcal bone lesion were nonspecific.^2^ There are reports in which vertebral cryptococcosis may mimic tuberculosis^3^ or malignancy^2^ on radiological finding. We are reporting a rare case of disseminated cryptococcosis where *Cryptococcus neoformans* was isolated from two different sites: sternum and psoas abscess involving the lumbar vertebra.

## CASE REPORT

**Figure 1:**
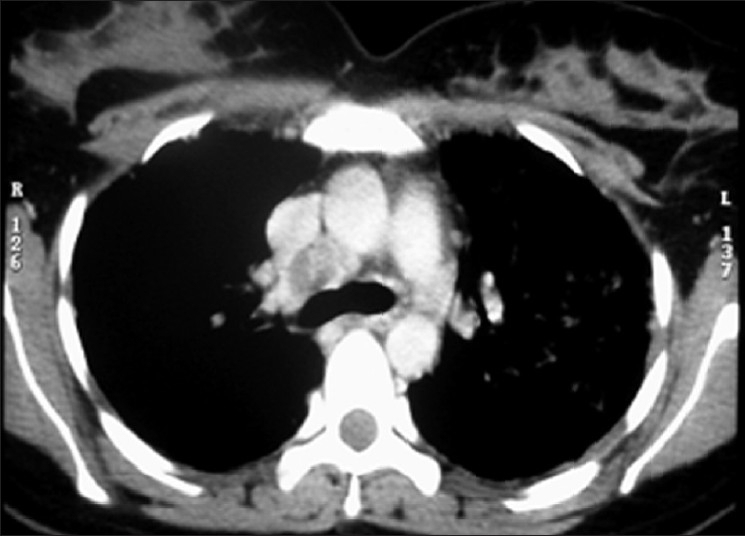
Contrast-enhanced computerized tomography of the thorax showing mediastinal lymphadenopathy and multiple small nodules in the right middle zone

**Figure 2:**
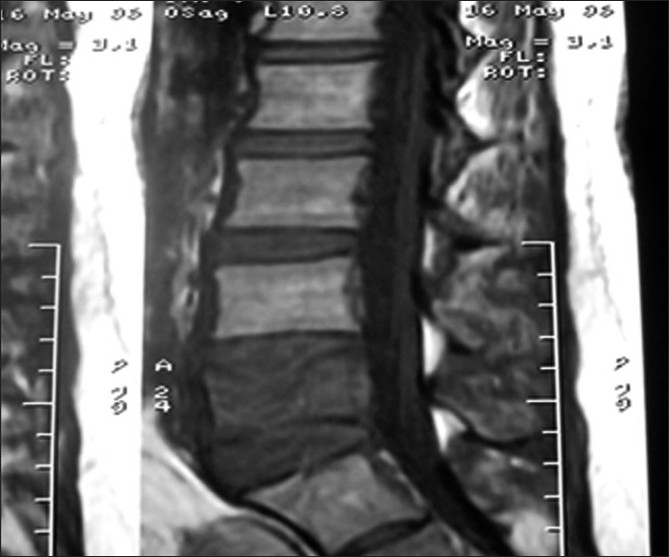
T1W MRI mid sagittal scan showing destruction of the L5 vertebra

**Figure 3:**
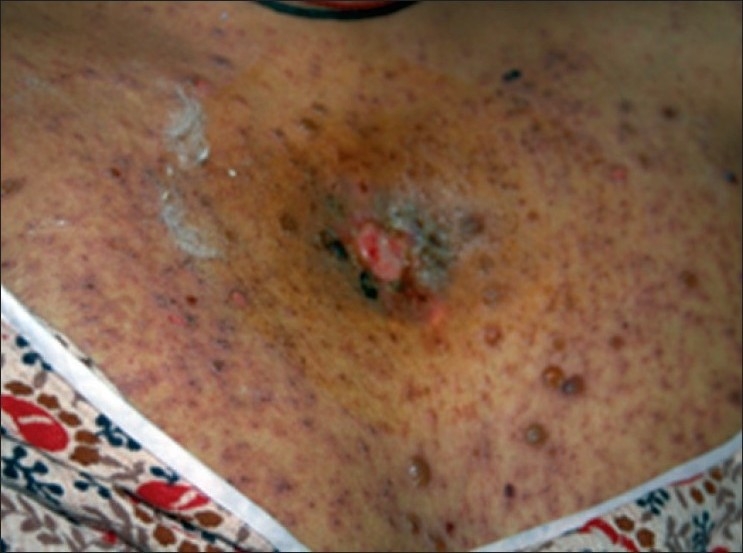
Clinical photograph of the same patient showing ulcerated lesion over the sternum with varicella rashes

A 29-year-old female presented with a history of low-grade fever (101–102°F) for the last 3 months in, December 2005. The fever increased in the evening, with mild nonproductive cough, and responded to antipyretics. There was no history of weight loss or loss of appetite. She experienced first-trimester spontaneous abortion for five times in each successive year since 2001. There was no past history of tuberculosis, diabetes, alcohol abuse and trauma. Fever did not resolve by various groups of antibiotics. She was investigated for the following laboratory tests: hemoglobin, 10.2 g%; total leukocyte count, 10,800/mm^3^; differential leukocyte count, N_78_L_19_E_3_M_0_; erythrocyte sedimentation rate, 110 mm/h; peripheral smear, negative for malaria; Widal test, negative; chest roentgenograph, normal; urine culture for bacteria, negative; Montoux test, 13 mm; ultrasound abdomen, normal. Contrast-enhanced computerized tomography (CECT) of the chest revealed right paratracheal lymphadenopathy and multiple small nodules in the right middle zone [Figure 1]. A diagnosis of pulmonary tuberculosis was made and the patient was put on antituberculosis treatment (ATT). She revisited in May 2006 with complaints of reappearance of fever. She was pregnant at that time. She also complained of backache and swelling over the sternum. Roentgenograph of lumbar spine was normal whereas magnetic resonance imaging (MRI) showed destruction of the L5 vertebra [Figure 2]. A diagnosis of suspected pott’s spine was made and ATT was changed to modified ATT, with the addition of ethionamide and cycloserine. She again had first-trimester spontaneous abortion. Her serum sample was negative for hepatitis B surface antigen, HIV I and II, antinuclear antibody and Venereal Disease Research Laboratory (VDRL). Immunoglobulin M antibodies for toxoplasma, rubella, cytomegalovirus and herpes were negative. CD4 count was 420/mm^3^ and the ratio of CD helper cells to CD suppressor cells was 1.41. Fine needle aspiration cytology (FNAC) of the swelling over the sternum was negative for acid-fast bacilli but reported capsulated budding yeast cells in the histopathology examination. The specimen was also sent to the mycology laboratory for fungal culture. 10% Potassium hydroxide (KOH) mount showed 4–6 *µ*-sized yeast cells with narrow neck budding. India ink preparation of the sample showed capsulated yeast cells. The sample was subjected to culture in multiple brain heart infusion agar (BHIA) tubes (BHIA plain and BHIA with Gentamicin). The culture isolate was identified as *Crytococcus neoformans*. Latex agglutination test for cryptococcal capsular polysaccharide antigen was positive from serum sample. Intravenous Amphotericin B 50 mg once a day was added in the treatment regime. The nodule over the sternum region ulcerated. Subsequent CECT chest and abdomen in July 2006 showed decrease in the size of pulmonary and paratracheal nodules as compared with the previous CECT, and also revealed destruction of sternal bone and formation of psoas abscess. Ultrasound-guided FNAC from psoas abscess was performed. No acid-fast bacilli were detected and bacterial culture was sterile from the psoas abscess aspirate. *Cryptococcus neoformans* was isolated from the sample. The patient was diagnosed as disseminated cryptococcosis with tuberculosis. Blood, urine and sputum samples for fungal culture were negative for *Cryptococcus neoformans*. She developed rashes all over the chest and extremities, which was diagnosed clinically as varicella infection [Figure 3] in the later part of July 2006. She was given intravenous acyclovir. She had a seizure attack on the fourth day of acyclovir treatment and then expired because of cardiac failure. Her postmortem bronchial aspirate and tissue biopsy of the ulcerated lesion over the sternum were negative for *Cryptococcus neoformans*.

## DISCUSSION

Osseous involvement occurs in 5–10% of patients with disseminated cryptococcosis. Cryptococcal osteomyelitis is a rare complication of disseminated cryptococcosis, and the vertebraes are the most common site of this infection. Most cases of cryptococcal osteomyelitis occur in immunocompromised patients. Cryptococcal osteomyelitis with or without other sites of infection in immunocompetent patients is less frequent.^2^ It is spread through the hematogenous route from the pulmonary focus or the lymph node, but direct inoculation through the skin and contiguous spread are also possible.^4^ Diagnosis is made from culture or histopathology report of tissue. The radiological findings and clinical features of cryptococcal bone lesion were nonspecific.^2^ There are reports in which vertebral cryptococcosis may mimic tuberculosis^3^ or malignancy^2^ on radiological finding. The diagnosis of cryptococcosis was delayed in this case because the patient was diagnosed as a case of pulmonary tuberculosis and clinical signs and symptoms and radiological findings in both the conditions are similar. The infection from the vertebra can spread along the anterior longitudinal ligament and lead to psoas or paravertebral abscesses.^5^ Psoas abscess developed in our case.

In this case, the patient conceived and resulted in spontaneous abortion. Ely *et al*.^6^ observed that there is an increased chance of dissemination of cryptococcosis during pregnancy. It may be due to decreased cell-mediated immunity in pregnancy.^7^ In the present case, pregnancy probably acts as a predisposing factor, which resulted in dissemination of *Cryptococcus neoformans*. It is also unclear whether there is any real increased risk of spontaneous abortion in pregnancy with cryptococcosis.^6^ In our case, the patient experienced spontaneous abortion. But, she had five such episodes previously and hence the sixth spontaneous abortion cannot be attributed to cryptococcosis. This case supports the finding that there is increased chance of dissemination of cryptococcosis in pregnancy. There is increased morbidity and mortality in varicella infection in pregnancy.^8^ Our patient developed varicella infection and expired even on intravenous acyclovir. Because the patient was on an antifungal agent, her postmortem samples were negative for *Cryptococcus neoformans*.

The patient was treated with Amphotericin B as an antifungal agent. Combination therapy with Amphotericin B and Flucytosine is considered for cryptococcal meningitis and severely ill cases of disseminated cryptococcosis. Amphotericin B alone is also used with success. Oral Fluconazole is suitable for treating the patient on an outpatient basis.^2^

This is an unusual case of disseminated cryptococcosis involving the sternum and lumbar vertebra, with the formation of psoas abscess with pulmonary tuberculosis in an HIV-negative immunocompetent patient. The vertebral cryptococcosis should be kept as a differential diagnosis in the clinical picture of pott’s spine.
